# Population genetics show that aphids (Hemiptera: Aphididae) are limited by summer host-plant distribution at the regional scale

**DOI:** 10.1093/jisesa/ieaf082

**Published:** 2025-10-06

**Authors:** Dion Garrett, Graham Teakle, Rosemary Collier, James R Bell, Ramiro Morales-Hojas

**Affiliations:** Rothamsted Insect Survey, Rothamsted Research, West Common, Harpenden, UK; Warwick Crop Centre, Wellesbourne Campus, University of Warwick, Warwick, UK; Warwick Crop Centre, Wellesbourne Campus, University of Warwick, Warwick, UK; Warwick Crop Centre, Wellesbourne Campus, University of Warwick, Warwick, UK; Rothamsted Insect Survey, Rothamsted Research, West Common, Harpenden, UK; Rothamsted Insect Survey, Rothamsted Research, West Common, Harpenden, UK

**Keywords:** population structure, currant-lettuce aphid, migration, host plant range, Asteraceae

## Abstract

*Nasonovia ribisnigri* (Mosley) is a severe aphid pest of outdoor lettuce, and the combination of sporadic and unpredictable colonization on outdoor lettuce, along with the breakdown of cultivar resistance, has left few effective control methods. The population structure (spatially and temporally) of *N. ribisnigri* is currently unknown in England, and therefore microsatellite markers were designed to estimate the impacts of host plant selection pressure (including host plant resistance) and environmental change. Biological samples collected between 2003 and 2020 from 10 sites across England were typed with microsatellite markers. The analysis of 8 microsatellites indicated a clear east-west divide between *N. ribisnigri* populations, which corresponds with current outdoor lettuce cultivation distribution in England, one of the aphid’s summer hosts. Analysis of gene flow indicated that aphids did not leave the eastern region; instead, there was strong evidence for aphids migrating from the West into the secondary host eastern region, possibly from the winter host (*Ribes* spp.) in Spring. This result suggests that although *N. ribisnigri* has the potential for long-distance migration, strong ties to the summer host (lettuce) determine migratory behavior at the population level. *N. ribisnigri* are mostly holocyclic and show a high level of inbreeding. Long-term trends revealed relatively stable populations, despite a recent breakdown of host plant resistance and other environmental changes, including favorable temperatures. The geographic and temporal structure of the *N. ribisnigri* population is discussed in relation to future pest management strategies.

## Introduction

Studies on population genetics can inform the scale at which pest populations should be managed (Catchen et al. 2017, Dusfour et al. 2019, Pu et al. 2020). For aphids, a key pest of most farming systems, microsatellite markers have been used to show the expansion of host range (Peccoud et al. 2008, Harrison and Mondor 2011), quantify the level of gene flow between populations (Orantes et al. 2012), elucidate host plant specialization and biotypes (Via and Hawthorne 2002, Vialatte et al. 2005), and the evolution of asexual and sexual morphs (Simon et al. 1999, Halkett et al. 2005). The information obtained can provide vital information on aphid migration, dispersal, host plant variation, and the evolution and spread of host plant resistant biotypes to inform on future mitigation strategies. Lifecycle plasticity is key to understanding why aphids are pests, and aphids are particularly well adapted to overcome high selection pressures that insecticides and host plant resistance impose on their populations (Loxdale et al. 2020). Aphids can utilize a combination of asexual and sexual reproduction types, have complex host plant and predator/prey interactions, and have the ability to disperse over large distances, all of which contribute to their success (Roitberg et al. 1979, Sunnucks et al. 1997, Simon and Peccoud 2018). These factors are especially important for aphids that are difficult to forecast and have sporadic outbreaks in crops, with limited means of control.

The currant-lettuce aphid, *Nasonovia ribisnigri* (Mosley) (Hemiptera: Aphididae), is the most damaging pest aphid on outdoor lettuce crops in the United Kingdom, continental Europe, America, and New Zealand (Reinink and Dieleman 1993, Stufkens and Bulman 2002, Nebreda et al. 2004, Sauer-Kesper et al. 2011). It is a heteroecious species, host-alternating between currant species (*Ribes spp.*), used during the winter/spring, and members of the Asteraceae family, which include lettuce (*Lactuca sativa*, Linnaeus) during the summer and autumn. Although *N. ribisnigri* is holocyclic, it is considered to be anholocyclic in temperate regions such as Spain (Nieto Nafria 1974). Outdoor lettuce crops can suffer significant contamination due to the presence of *N. ribisnigri* in the lettuce head, where the aphids are inaccessible to foliar applications of insecticides that do not have systemic activity (Natwick and Lopez 2016). The use of systemic neonicotinoids was one of the strategies incorporated by conventional outdoor lettuce growers to reduce the impact of *N. ribisnigri*, but the subsequent ban on their use in the UK for non-flowering crops left lettuce growers with few management options (DEFRA 2021).

To address the issues concerning insecticides, a lettuce cultivar that had proven resistance to *N. ribisnigri* was introduced to the growers’ recommended list during the early 1980s (Garrett et al. 2024). It was discovered that a single gene (*Nr-gene*) in these lettuce cultivars was responsible for conferring near-complete resistance to *N. ribisnigri* and was subsequently incorporated into several outdoor lettuce growers, prominently situated in the East of England (Tatchell et al. 2017). Due to few alternative control strategies for *N. ribisnigri* on outdoor lettuce, these cultivars were relied upon heavily during the summer months, and, as a result, resistant-breaking *N. ribisnigri* biotypes were identified that could overcome the *Nr-gene* in the lettuce host plant (van der Arend 2003). The first reports of resistant-breaking biotypes occurred in France and Germany during 2007, with the first reports of this biotype being found in Kent, United Kingdom, during 2009 (Hough 2013). The implications of this high selection pressure can significantly affect the genetic structure of insect populations (Hawkins et al. 2019). For example, the high selection pressure of the *Nr-gene* in outdoor lettuce would lead to an increase of resistant alleles in *N. ribisnigri* populations and a decrease in frequency of susceptible alleles.

Using standardized methods to capture aphids allows for forecasting models to be constructed that help predict the likelihood of an aphid outbreak occurring in crops (Harrington and Woiwod 2007, Thackray et al. 2009). The main methods employed for detecting and monitoring aphids are the use of yellow sticky traps, yellow water traps, and suction traps (Taylor 1962, Lamichhane et al. 2016). Paradoxically, whilst large numbers of *N. ribisnigri* are quick to colonize outdoor lettuce crops in the summer, winged adults rarely appear in many insect traps, including the network of 16 12.2 m suction traps used to monitor aphids (RIS 2022). Interestingly, *N. ribisnigri* does not typically become a problem on outdoor lettuce until mid-to late summer. Most host-alternating aphids produce alates that migrate to a summer host (including crops) during the spring. Thus, there exists a gap in *N. ribisnigri* occurrence from spring migration to crop infestation. The combination of delayed colonization in summer and lack of alates in current trapping techniques produces inaccuracies in the forecast of activity and subsequent crop colonization by *N. ribisnigri*.

Despite its pest status in the United Kingdom, continental Europe, America, and New Zealand, the level of gene flow and population structure of *N. ribisnigri* has yet to be studied. Using microsatellite markers developed based on a newly assembled genome (Garrett et al. 2024), we analyzed the structure of the English population using archived samples of aphids captured over 2 decades in the network of suction traps run by the Rothamsted Insect Survey. This study aimed to (i) quantify the level of gene flow between populations in England and infer dispersal potential; (ii) understand whether populations have evolved as a response to environmental change, both spatially and temporally; (iii) determine whether changes in population structure could be attributed to the development of biotypes that were able to overcome the resistance conferred by the *Nr-gene*, which was present in some lettuce crops from 2008 onwards; and (iv) determine the status of anholocyclic reproduction. The information obtained will provide important ecological and evolutionary information on a species of pest aphid that is poorly understood and considered to be the most damaging to outdoor lettuce in many parts of the world. It is expected that this study will deliver insights to inform future pest management strategies as well as an understanding of the past and current demographics of *N. ribisnigri*.

## Materials and Methods

### Study Organism and Site Locations

All *N. ribisnigri* used in this study were collected between 2003 and 2020 from the Rothamsted Insect Survey’s (RIS) network of 12.2 m suction traps in the United Kingdom. Aphids caught in the suction traps were identified to species using the taxonomic key in Blackman and Eastop (2000) and stored in a 100% ethanol:glycerol solution at a ratio of 95:5 in a sample archive. A total of 531 *N. ribisnigri* from 10 sites in England (Supplementary Fig. S1) were removed from the archive and then stored in 100% ethanol at −20 °C before analysis. Due to the inherently low incidence of *N*. *ribisnigri* in the traps, the criteria for site selection were that there was more than one individual per site-year and that all the aphids were caught between April and August (this is typical migration from winter to summer host [including outdoor lettuce]). Because aphids are small, soft-bodied insects that can be damaged easily during the collection and storage process, only undamaged (fully intact) *N. ribisnigri* were used, thus avoiding a low yield of DNA and cross-contamination.

### DNA Extraction

DNA extractions were conducted on whole individual aphids using the Qiagen QIAmp DNA Micro Kit (Qiagen, Manchester, United Kingdom) following manufacturer’s recommendations, with the following adjustments: individuals were homogenized using a pestle in liquid nitrogen in a 1.5 ml Eppendorf, 180 µl of ATL buffer was added along with 20 µl of proteinase K, mixed with gentle flicking, briefly spun down using a microcentrifuge, and incubated overnight on an orbital shaker at 200 to 220 rpm at 56 °C. 200 µl of AL buffer (with 1 µg/µl RNA carrier) was added to the samples the following morning, which were inverted 10 times, then 200 µl of 100% ethanol was added and the samples were incubated at room temperature for 5 min. Each entire sample was transferred to a QIAmp DNA Micro Kit spin column and spun at 8,000 rpm (6,010 g) for 1 min at room temperature. The flow-through was discarded, and the column was placed into a new collection tube, with 500 µl of AW1 buffer added, and centrifuged at 8,000 rpm. The flow-through was again discarded and placed in a new collection tube with 500 µl of AW2 buffer added into the column and centrifuged at 8,000 rpm for 1 min at room temperature. Finally, the flow-through was again discarded, the column was added into a new collection tube, and spun at 13,000 rpm (15,871 g) for 3 min at room temperature. The column was placed into a 1.5 ml Eppendorf, and 40 µl of AE buffer was added directly onto the column membrane. It was then incubated at room temperature for 10 min and spun down for 1 min at 14,000 rpm (18,440 g). To improve DNA yield, the recovered AE buffer was placed again onto the column membrane, and the process was repeated following the same procedure. All DNA samples were quantified using the Qubit dsDNA HS Assay Kit (ThermoFisher Scientific) following manufacturer’s guidelines, and DNA was stored in a −20°C freezer after quantification, prior to PCR amplification and downstream analysis. A negative control of molecular biology grade H_2_O was included in the DNA extraction step.

### Microsatellite Discovery

The reference genome for *N. ribisnigri* (biotype—Nr8, BioProject ID: PRJNA857679) was used to search for suitable microsatellite markers. A total of 4,778 scaffolds were uploaded into Msatcommander 0.8.2 (Faircloth 2008) for the detection of microsatellites following the set parameters used previously and described in the literature (Jun et al. 2011) (Supplementary Table S1). All microsatellites discovered (including flanking regions) were selected manually and extracted for downstream primer design from the *N. ribisnigri* genome (Nr8) using Geneious 10.1.3 software (https://www.geneious.com). Microsatellites located near to the end of sequence scaffolds were not used due to read coverage and read quality dropping inherently. In addition, to avoid marker-biases from potentially non-neutral markers, only microsatellites that were not closely linked to functional genes were used (Hung et al. 2016). Forward and reverse primers were designed for each microsatellite in the flanking regions using Primer3 (http://bioinfo.ut.ee/primer3-0.4.0/). A total of 68 microsatellites were selected for PCR (Polymerase Chain Reaction) amplification and sample validation. Microsatellite primers were obtained from Sigma-Aldrich.

### Validation, PCR Amplification and Sequencing of Microsatellites

Seven *N. ribisnigri* cultures were used to validate the identified microsatellite markers. Their successful amplification and the polymorphism level of each locus were verified using PCR amplification. All 7 cultures were provided by Warwick Crop Centre from long-established colonies of *N. ribisnigri*. These were taken to Rothamsted Research, where isogenic lines of each culture were established (Supplementary Table S2). Isogenic lines were established by selecting one apterous (founding mother) from each colony and placing it onto a new *L. sativa* (cv. Pinokkio) host plant in new, separate insect rearing chambers. The rearing chambers were clear acrylic cylinders (45 cm height and 15 cm diameter) with holes and fine mesh for respiration and had both a lid and bottom to contain a whole lettuce plant in a pot hosting the *N. ribisnigri* culture. Once a founding mother had produced 5 to 10 nymphs, they were removed, and the new isogenic culture was left to establish for 7 to 14 d. Each culture was reared on a lettuce (cv. Pinokkio) plant at 20 °C, 16:8 h (light:dark) in individual rearing chambers.

Amplification reactions were performed in a total volume of 10 µl containing 1 µl of 10 ng/µl of DNA, 5 µl of primer master mix (ThermoFisher Scientific, Bishop’s Stortford, Hertfordshire, United Kingdom), 3.55 µl of molecular-grade water, 0.2 µl of M13 Tail SSR (FAM), 0.05 µl of M13 forward-tailed primer (fluorescent labelled dyes; 6-FAM, VIC, NED, and PET [ThermoFisher Scientific]), and 0.2 µl of reverse primer. All reactions were run on a thermocycler with amplification cycle: 94 °C for 1 min; 30 cycles of 94 °C for 40 s, annealing at each locus optimal temperature for 1 min, 72 °C for 30 s; 8 cycles of 94 °C, 56 °C for 1 min, 72 °C for 30 s; hold 20 °C. The annealing temperature was optimized for each microsatellite marker with 2 °C increments and ranged between 50 and 58 °C (Supplementary Table S3). Amplification products were run on an ABI Prism 7,500 sequencer (Applied Biosystems), (Warrington, Cheshire). For this, 1 µl of the PCR product was added to 8.8 µl Hi Dye Formamide (Applied Biosystems) and 0.2 µl Liz 500 Size Standard (ThermoFisher Scientific) and incubated at 94 °C for 6 to 10 min on a thermocycler for denaturation. Immediately after the cycle, samples were transferred to ice for rapid cooling and sequenced immediately. The amplicons generated were sequenced individually using the ABI-3100 sequencer (Applied Biosystems). Microsatellites were scored using the microsatellite plugin for Geneious. Microsatellite analysis was conducted following the recommendation in the online tutorial www.geneious.com/tutorials/microsatellites. In brief, each microsatellite marker for every sample was inspected to confirm the Liz 500 size standard was called correctly and manually adjusted if not. The 6-FAM fluorescent dye was used for microsatellite validation only, with the remaining 3 fluorescent dyes (VIC, NED, and PET) included in the population genetic the analysis experiment (Supplementary Table S4). Each microsatellite was visually inspected for peaks, and any peaks present that were within range (eg 6-FAM range is 160 to 450 bp) but not called within the Geneious microsatellite software, were corrected. Once peaks were called, bins were predicted for each sample based on the observed peaks and their size, using the integrated sizing algorithm in Geneious. After this step, an allele table was generated, which contained the microsatellite peaks and their size, and exported in a CSV file for further analysis. A selection of 8 microsatellites was selected based on their motif repeats (di- and trinucleotides) and incorporated into the final analysis. due to the limited quantities of DNA obtained from some of the *N. ribisnigri* samples.

### Population Analysis

To study the genetic differences between populations before and after the breakdown in host plant resistance to *N. ribisnigri*, first recorded in England in 2009, samples were grouped into 3 categories (2003 to 2007, 2008 to 2014, and 2015 to 2020) (Table 1). Between 2003 and 2007, the resistance-breaking biotype was not present in the UK but was detected in France and Germany in 2007 and subsequently in 2009 in the UK. By selecting the 2003 to 2007 grouping reduces the chances of including resistant-breaking biotypes that may have appeared 1 to 2 yr prior to their detection. The second grouping (2008 to 2014) was used to determine whether a difference in population structure could be observed up to 6 yr after the breakdown in host plant resistance. The third grouping (2015 to 2020) was used to understand whether any other longer-term changes in population structure had occurred. Additionally, separating the analysis into 3 periods of similar length provided a relatively even distribution of samples for analysis. Each site-year grouping contained a minimum of 4 individuals. A total of 146 aphids (*N. ribisnigri*) were studied over 10 locations across England (Supplementary Fig. S2).

**Table 1.** Number of *Nasonovia ribisnigri* individuals collected from the Rothamsted Insect Survey (RIS) archive and used in the current study. The suction trap at York was not established pre 2008, and therefore no samples were collected

Sites	2003 to 2007	2008 to 2014	2015 to 2020	Total (2003 to 2020)
Brooms Barn	6	4	4	14
Hereford	5	5	4	14
Kirton	4	4	6	14
Preston	4	5	4	13
Rothamsted	5	4	6	15
Starcross	6	5	4	15
Wellesbourne	8	4	9	21
Writtle	6	4	4	14
Wye	4	6	4	14
York	-	5	7	12
**Total**	48	46	52	146

Observed and expected heterozygosity (*H_O_* and *H_E_*) were obtained using the R packages *ade4* (v.1.7-16) and *adegenet* (v.2.1.3) (Jombart 2008). A Principal Component Analysis (PCA) was performed to visualize any structure signal in the microsatellite data for all sites using the R packages *adegenet* and *ade4* (s.class function). A Bartlett test of homogeneity of variances was conducted to test whether the mean observed heterozygosity was significantly lower than the mean heterozygosity (R package: *Stats* [v.4.0.3]) (RStudio Team 2020). A subsequent paired t-test was incorporated to determine whether the Bartlett test was significant (α = 0.05).

Structure software (v.2.3.4) (Pritchard et al. 2000) was implemented using a Bayesian admixture model, with no population assumed *a priori*, to identify genetically homogenous groups within the genotyped data set (Pritchard et al. 2000). The K values tested ranged between 1 and 10, with 10 simulations for each. Structure runs were set up with 100,000 burn-in iterations with 500,000 Markov chain Monte Carlo (MCMC) repetitions to ensure convergence was achieved. The Evanno method was used to detect the number of clusters using an ad hoc statistic (Δ*K*) based on the rate of change in the log probability of data between successive *K* values (Evanno et al. 2005). To identify and visualize the number of genetic clusters within the different groups, the R package *pophelper* was used (Francis 2017).

A hierarchical analysis of molecular variance (AMOVA) was conducted using the software Arlequin (v.3.5.2.2) to investigate genetic variation among populations (Excoffier et al. 2005). The parameters were set for 10,000 permutations, with the gametic phase set to unknown and codominant data. The AMOVA was used to test the population structure for: (i) differentiation between all sites (*n *= 10) and all years (2003 to 2020), (ii) all sites and year grouping (2003 to 2007), (iii) all sites and year grouping (2008 to 2014), (iv) all sites and year grouping (2015 to 2020), and (v) differentiation between West (Hereford, Preston, Starcross, and Wellesbourne) and East (Brooms Barn, Kirton, Rothamsted, Writtle, Wye, and York) between all years (2003 to 2020) and year groupings (2003 to 2007, 2008 to 2014, and 2015 to 2020). York was excluded from both the hierarchical AMOVA and pairwise *F_ST_* analysis as no samples were taken during 2003 to 2007, as the suction trap was installed in 2008. Comparisons between sample locations were also computed, and pairwise *F_ST_* values obtained (10,000 permutations and significance set to 0.05). Pairwise *F_ST_* information was extracted from Arlequin (10,000 permutations) into RStudio and plotted using the R packages *XML* (v.3.99 to 0.5), *corrplot* (v.0.84), *magrittr* (v.2.0.1), and *dplyr* (v.1.0.2). A Mantel test was conducted in Arlequin using 10,000 permutations to identify whether there was a correlation between geographical distance and genetic distance (*F_ST_*). Latitude and longitude were obtained from the RIS suction trap network database.

## Results

### The Population Structure of *Nasonovia Ribisnigri*

**Fig. 1:**
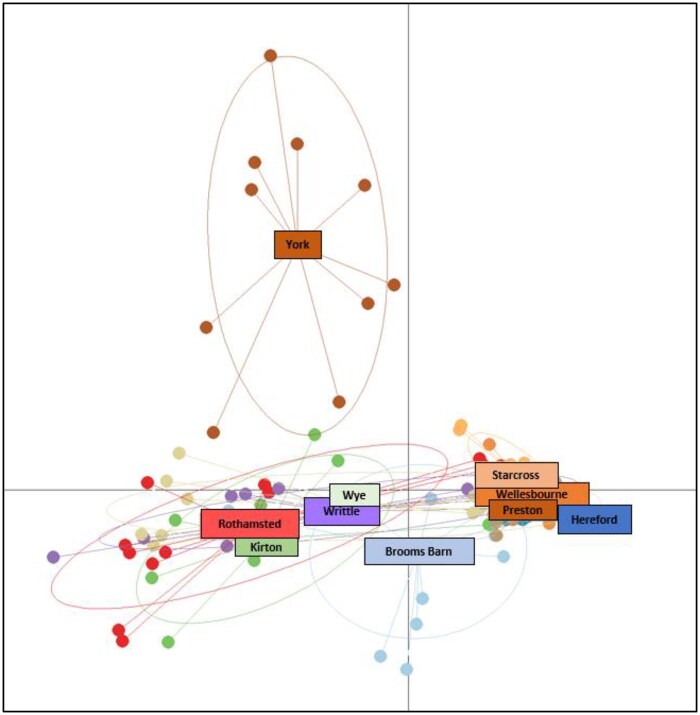
Principal component analysis of the microsatellite variation in the currant-lettuce aphid (*Nasonovia ribisnigri*). Loose groupings of the West population (Hereford, Preston, Starcross, and Wellesbourne) and the East (Brooms Barn, Kirton, Rothamsted, Writtle, and Wye), with York forming a separate group, can be seen. However, this third subgroup was not supported in further analyses.

**Fig. 2:**
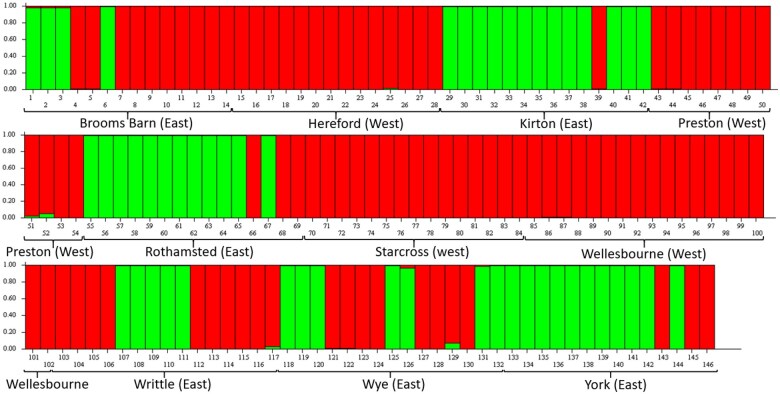
Genetic structure bar plot inferred using Bayesian analysis in Structure software with 2 clusters (*K *= 2). Each bar represents an individual, with the color of the bar representing the likelihood of membership to either population cluster. A grouping of 2 was determined to be most likely, despite the out grouping of York, highlighted in the principal component analysis. Parameters used in structure: 100,000 burn-in steps and 500,000 Markov Chain Monte Carlo (MCMC) steps.

Out of 531 samples of *N. ribisnigri* collected between 2003 and 2020, only 146 samples (27.5%) yielded concentrations of DNA over the 5 nl/µl threshold required to conduct the population genetic analysis (Supplementary Fig. S2). The principal component analysis (PCA) biplot showed 3 groups of *N. ribisnigri*: Group 1 (Hereford, Preston, Starcross, and Wellesbourne), Group 2 (Brooms Barn, Kirton, Wye, Writtle, and Rothamsted), and a potential third group (Group 3), which includes York only (Fig. 1). However, the structure analysis results suggested that the most likely number of genetic clusters was *K *= 2, as the result for *K *= 3 was low (Evanno et al. 2005). Unfortunately, this method does not estimate Δ*K* for *K *= 1, but since Mean L (*K*) is not maximized for *K *= 1, this suggests that the number of clusters is 2 (Morales-Hojas et al. 2020a) (Fig. 2). These 2 population groups loosely correspond to an east-west divide between sample sites. However, some individuals from the western cluster were present at some of the sites in the East, whereas no individuals from the eastern populations were present at sites in the West. Additionally, the lack of admixture in the barplots of the structure analysis supports the conclusion that there is a lack of interbreeding between the 2 clusters.

**Fig. 3:**
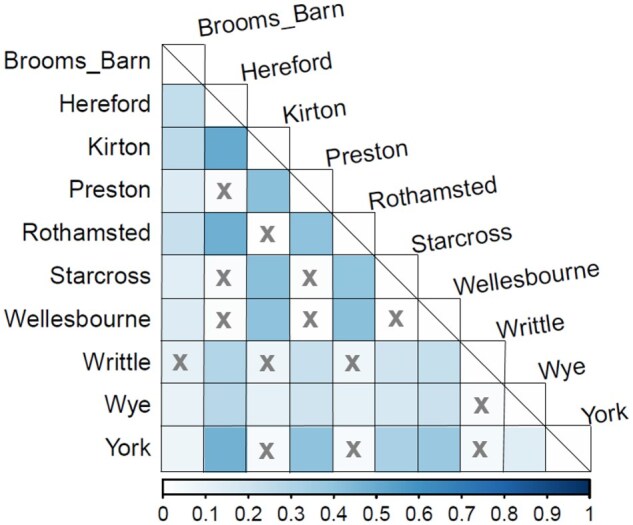
Population pairwise *F_ST_* of *Nasonovia ribisnigri* between population sites between 2003 and 2020 showing the level of genetic differentiation. The scale bar on the *x*-axis highlights the pairwise *F_ST_* value, the darker the square, the higher the *F_ST_* value. X indicates no significant *F_ST_* value between 2 populations (significance set to *F_ST_* >0.20, *p* = < 0.05). Top 3 highest *Fst* values: Hereford and Kirton (0.52), Hereford and Rothamsted (0.49), and Hereford and York (0.47).

The population genetic differences between sites, estimated by pairwise *F_ST_* for *N. ribisnigri*, ranged from moderate to high (Fig. 3). The highest genetic differentiation (*F_ST_*) was observed between the East and West populations. Despite their relatively close geographical location, Hereford and Wellesbourne (West) exhibited a high level of genetic differentiation from Rothamsted (East). Broom’s Barn exhibited a low *Fst* between all other sites except Writtle. The Mantel test identified a significant correlation between geographical distance and genetic distance for East and West, which suggests that the differentiation is partly a result of isolation by distance (*F_ST_* = 0.307, *p *< 0.001).

The observed heterozygosity for all sites was lower than the expected heterozygosity (*H_O_* = 0.29, *H_E_* = 0.55), with an inbreeding coefficient (*F_IS_*) of 0.64 (*p *< 0.001) (Table 2). A total of 70.8% of individual loci from the eastern population (Brooms Barn, Kirton, Rothamsted, Writtle, Wye, and York) had expected and observed heterozygosity that were significantly different, whereas the western population (Hereford, Preston, Starcross, and Wellesbourne) had only 50% (Table 2). The total *H_O_*, averaged between individual microsatellite loci and sites, was lower in the eastern genetic cluster (0.27) compared to the West (0.32). The *H_O_* between both East and West populations overall was similar; however, the H_O_ for York was much lower than for all sites (0.09), which reduced the average *H_O_* for the East region. For all sites, mean *H_O_* was lower than *H_E_*, and the inbreeding coefficient (*F_IS_*) was above 0 and significant.

**Table 2.** Genetic diversity estimates for the 10 sites, combined sites; and East and West populations of *Nasonovia ribisnigri* between 2003 to 2020

Site	*n*	*H_O_*	*H_E_*	*F_IS_*	p*HWE*
Broom’s Barn	14	0.29	0.69	0.69	3
Hereford	14	0.3	0.43	0.40	0
Kirton	14	0.23	0.51	0.80	2
Preston	12	0.39	0.47	0.47	0
Rothamsted	15	0.3	0.59	0.63	3
Starcross	15	0.23	0.49	0.69	1
Wellesbourne	22	0.37	0.5	0.43	2
Writtle	14	0.33	0.63	0.78	5
Wye	14	0.36	0.62	0.63	4
York	12	0.09	0.63	0.94	5
All	146	0.29	0.55	0.64	25
East	83	0.27	0.61	0.74	22
West	63	0.32	0.47	0.49	3

*n—*number of individuals, *H_O—_*mean observed heterozygosity for all 8 microsatellite loci, *H_E—_*mean expected heterozygosity for all 8 microsatellite loci, *F_IS—_*population specific inbreeding coefficient, and p*HWE—*number of loci for each population which departed from Hardy-Weinberg equilibrium (HWE).

There were no deviations from the HWE in most of the populations except for 3 during 2015 to 2020 (Kirton, Rothamsted, and York). A total of 8.5% (25) of the individual loci deviated from the HWE in all populations (*n *= 146), with the majority of the deviations (13.2%) occurring in the East (*n *= 63) and 2.4% (3) deviations in the West (*n *= 63). York had the lowest observed heterozygosity of the sites (0.09) and subsequently the highest inbreeding coefficient (*F_IS_* = 0.94, *p *< 0.001). However, the H_E_ was similar to the other populations, and this suggests that an equal range of genetic diversity was sampled from each site.

AMOVA analysis indicated that most of the genetic variation is between the East and West grouping, accounting for 30.14% of the genetic variation of these 2 clusters over (*F_CT_* = 0.30, *p *< 0.001) (Table 3). The amount of variation among populations within the East and West groups was low (5.67%) but significant (*F_SC_* = 0.081, *p *< 0.001). The remaining variation was found among individuals within populations (23.64%, *F_IS_* = 0.368, *p *< 0.001) and within individuals (40.55%, *F_IT_* = 0.594, *p *< 0.001).

**Table 3.** Hierarchical analysis of molecular variance (AMOVA) for *N. ribisnigri*. Genetic variation of all individuals between the East and West groupings, as identified in the structure among all years (2003 to 2020)

Source of variation	df	Sum of squares	Variance components	% variation	Fixation indices	*p*
Among groups	1	81.91	0.552	30.14	*FCT* 0.301	<0.001
Among populations within groups	27	71.49	0.103	5.67	*FSC* 0.081	<0.001
Among individuals within populations	117	188.33	0.433	23.64	*FIS* 0.368	<0.001
Within individuals	145	108.5	0.743	40.55	*FIT* 0.594	<0.001

East geographical cluster comprises of Brooms Barn, Kirton, Rothamsted, Writtle, and Wye among all years (2003 to 2020). West geographical cluster comprises of Hereford, Preston, Starcross, and Wellesbourne among all years (2003 to 2020). df = degrees of freedom.

### 3.2 Temporal Analysis of Nasonovia Ribisnigri Populations

The hierarchical AMOVA for all sites, excluding York (*n *= 9), and among years for the 3-yr groupings (2003 to 2007, 2008 to 2014, and 2015 to 2020) was not found to have significant genetic differentiation (4.58%, *F_ST_* = −0.045, *p *= 0.98), possibly because of significant variation (28.19%, *F_SC_* = 0.269, *p *< 0.001) among individuals within groups (Table 4A). A similar amount of genetic variation (28.13%) was found among populations within the site-years (*F_IS_* = 0.368, *p *< 0.001), and the remaining 48.26% was attributed to within individuals (*F_IT_* = 0.517, *p *< 0.001).

**Table 4.** Hierarchical analysis of molecular variance (AMOVA) for *N. ribisnigri*. (A) Genetic variation of all individuals between 9 sites among year groupings (2003 to 2007; 2008 to 2014; 2015 to 2020); (B) Eastern geographical cluster determined by structure comprising of Broom’s Barn, Kirton, Rothamsted, Writtle, and Wye among year groups; (C) Western geographical cluster determined by Structure comprising of Hereford, Preston, Starcross, and Wellesbourne among year groups. York was excluded from temporal analysis, as the suction trap was installed in 2008, and therefore no data was collected between 2003 to 2007

Source of variation	Sum of squares	Variance components	% variation	Fixation indices	*p*
**(A) All**					
Among year groups	−1.107	−0.07	−4.58	*FCT* −0.045	0.98
Among populations within year groups	154.5	0.434	28.19	*FSC* 0.269	<0.001
Among individuals within populations	188.33	0.433	28.13	*FIS* 0.368	<0.001
Within individuals	108.5	0.743	48.26	*FIT* 0.517	<0.001
**(B) East**					
Among year groups	2.003	0.035	−3.06	*FCT* −0.030	0.73
Among populations within year groups	40.461	0.150	12.97	*FSC* 0.125	<0.001
Among individuals within populations	94.711	0.392	33.88	*FIS* 0.376	<0.001
Within individuals	54	0.65	56.21	*FIT* 0.437	<0.001
**(C) West**					
Among year groups	0.362	0.064	−5.57	*FCT* 0.055	0.98
Among populations within year groups	23.865	0.142	12.29	*FSC* 0.116	0.01
Among individuals within populations	62.058	0.136	11.76	*FIS* 0.126	0.01
Within individuals	59.5	0.944	81.53	*FIT* 0.184	<0.001

There was no significant difference between the 3-yr groupings in the eastern cluster, with only 3.06% variation accounted for (*F_CT_* = −0.030, *p *= 0.73) (Table 4B). Most genetic variation (56.21%) was within individuals (*F_IT_* = 0.437, *p *< 0.001), with the remaining 33.88% attributed to among individuals within populations (*F_IS_* = 0.376, *p *< 0.001). These results were similar in the West (Table 4C), with no significant genetic variation among different year groups (5.57%, *F_CT_* = 0.055, *p *= 0.98). The pairwise *F_ST_* values between years were small and non-significant, which further supports an absence of genetic variation (Table 5). This result was mirrored when populations were divided into the 2 separate clusters between the East and West.

**Table 5.** Pairwise *F_ST_* values for all individuals collected in different year groups (A), between individuals in the East (B), and individuals in the West (C). N shows the number of individuals in each group

	*n*	2003 to 2007	2008 to 2014	2015 to 2020
**(A) All**				
2003 to 2007	48	-		
2008 to 2014	38	−0.025	-	
2015 to 2020	48	−0.038	0.016	-
**(B) East**				
2003 to 2007	25	-		
2008 to 2014	19	−0.009	-	
2015 to 2020	27	−0.05	0.022	-
**(C) West**				
2003 to 2007	23	-		
2008 to 2014	19	−0.04	-	
2015 to 2020	21	0.023	−0.063	-

**Fig. 4:**
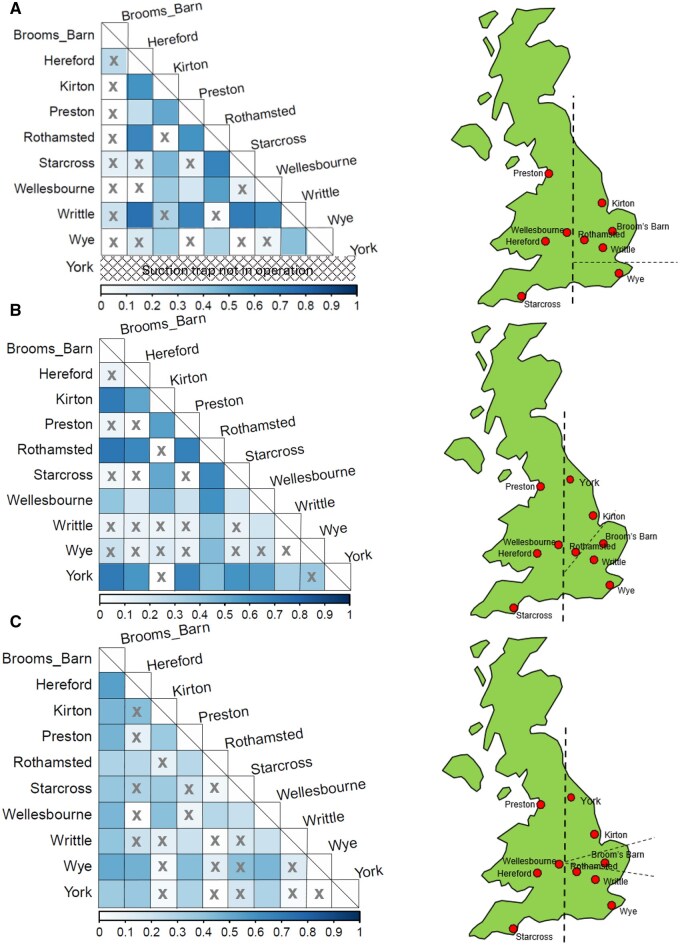
Population pairwise *F_ST_* of *Nasonovia ribisnigri* between population sites and separated between year groups showing the level of genetic differentiation: A) 2003 to 2007; B) 2008 to 2014; C) 2015 to 2020. The scale bar on the *x*-axis highlights the pairwise *F_ST_* value, and X indicates no significant *F_ST_* value. The UK map to the right of each pairwise *F_ST_* analysis indicates population site locations, with the thicker dashed line highlighting the East-West population divide (genetic barrier) and the thinner dashed line highlighting the main disparities between East year groupings and the general divides in the East population.

Overall, pairwise *F_ST_* highlights no significant change in the interpopulation genetic differentiation at each of the 3-yr intervals analyzed (2003 to 2007, 2008 to 2014, and 2015 to 2020), but some subtle disparities exist when examining individual population pairwise *F_ST_* between these sample points (Fig. 4). In particular, the Wellesbourne population during 2008 to 2014 had low but significant genetic differentiation from other populations in the western cluster and a moderate-high significant genetic difference from Rothamsted, despite being relatively close geographically (Fig. 4B). The aphids from Broom’s Barn captured during the period 2003 to 2007 had low genetic differentiation with respect to all sites (Fig. 4A), which is likely to be an artifact due to some microsatellite loci being absent during sequencing and analysis. Between 2015 and 2020, Broom’s Barn data showed significant pairwise differentiation (*F_ST_*) from other populations, and this indicates that this site had moderate genetic differentiation between these sample points (Fig. 4C).

The overall majority of pairwise differentiation in the East was low and non-significant (Supplementary Fig. S3A). In the West, the genetic differentiation between locations has remained relatively stable over the 18 yr, evidenced by the non-significant *F_ST_* values throughout (Supplementary Fig. S3B). The main genetically different group is Wellesbourne during 2008 to 2014, in which significant *F_ST_* values were detected between 0.15 and 0.26. Broom’s Barn was an exception and indicated moderate to high levels of genetic differentiation between sites during 2008 to 2014 and 2015 to 2020.

## Discussion

This study is the first to report the population genetics of *N. ribisnigri* using microsatellite markers developed from a recently assembled genome (Garrett et al. 2024). Two distinct genetic clusters were identified, which loosely correspond to an east-west divide in England. The PCA results highlighted that York was potentially a third cluster (Fig. 1), but this was not supported in the AMOVA (Tables 3 and 4) results nor the structure analysis (Fig. 2), which suggested that 2 groups were more likely. Additional samples would be required to confirm whether York is in fact a third unique cluster. Genetic differences between these 2 clusters account for 30.14% of the genetic variability throughout the sampling period, higher than for the bird-cherry oat aphid (*Rhopalosiphum padi*) population, at 17% (Morales‐Hojas et al. 2020a). It is unlikely that the 2 clusters corresponded to sexual (holocyclic) and asexual (anholocyclic) morphs due to the positive and significant inbreeding coefficient (F_IS_) observed in every population within both genetic clusters. The *F_IS_* population-specific inbreeding coefficients were higher in populations in the East compared to the West. Very high *F_IS_* levels were observed for York, Kirton, and Writtle, which could be a result of most of the sample collection occurring June to August for these sites. It is therefore likely that these aphids would have already migrated to their summer host and reproduced asexually from the same clonal linage, thus causing the high observed inbreeding coefficients (Blackman and Eastop 2000; Diaz and Fereres 2005). Given the generally low number of individuals sampled and the deficiency in observed heterozygosity in all populations, it is most likely that these *N. ribisnigri* are inbred. The lower-than-expected numbers of individuals used in the analysis have limited the chances of detecting alleles at low frequencies. More individuals would have provided a stronger temporal analysis. Unfortunately, due to the extremely low numbers caught in the Rothamsted Insect Survey (RIS) 12.2 m suction traps, combined with their small size and low DNA yield (27.7% >5 µl/ng), it was not possible to obtain further samples, as the majority of *N. ribisnigri* had already been collected in the present study.

The increasingly warm climate and generally milder winter conditions in northern Europe permit some aphid species to maintain an anholocyclic lifecycle and overwinter as immature nymphs or adult morphs (Williams and Dixon 2007). For example, anholocyclic peach-potato aphids, *Myzus persicae*, and English grain aphids, *Sitobion avenae*, are spreading further north in line with favorable meteorological conditions (Blackman 1974, Simon et al. 1999) and the potential spread of an insecticide resistance “superclone” (Morales‐Hojas et al. 2020b). It has been shown that *N. ribisnigri* typically overwinters on a woody host plant (eg blackcurrant, *Ribes nigrum*), but some research has shown that they are able to overwinter as a nymph or adult on herbaceous plants such as perennial sow thistle (*Sonchus arvensis*), chicory (*Cichorium inybus*), and corn speedwell (*Veronica arvensis*) (Hough 2013). Despite anecdotal evidence that *N. ribisnigri* is undergoing anholocyclic reproduction in the South, the results from this study suggest that populations of *N. ribisnigri* are mostly holocyclic, including the southernmost populations sampled in England, and typically overwinter as eggs. It is possible that anholocyclic populations of *N. ribisnigri* do exist in the South, but they were not detected in either southerly location or, if present, are likely to be small, isolated populations.

Over the 18-yr study period (2003 to 2020), there was no statistically significant difference between the 3-yr groupings in both the eastern and western clusters. This suggests that both the East and West clusters have not changed significantly over time, which is supported by the positive and significant inbreeding coefficient (*F_IS_*). However, the *N. ribisnigri* caught at Broom’s Barn showed significant pairwise differentiation (*F_st_*) from the other sites in the eastern cluster for both the second (2007 to 2014) and third (2015 to 2020) year groupings (Supplementary Fig. S3B). Although there is no official data on the distribution of outdoor lettuce in the UK, an agronomist from Fresh Produce Consultancy (with over 25-yr in the industry) has confirmed that areas used to grow outdoor lettuce in the UK have not significantly changed over the last 20-yr (Norman 2025). As a result, the subtle genetic structural changes are unlikely linked to a change in outdoor lettuce production in the UK. In addition, human-mediated transport of *N. ribisnigri* on lettuce may have influenced the shaping of the population structure but due to increased biosecurity measures and cultivation techniques, this is unlikely to have played a significant role. This change in *F_st_* level coincides with the first reports of resistance-breaking biotypes of *N. ribisnigri* in commercially grown lettuce (Thabuis et al. 2014). Farms in the areas surrounding the Broom’s Barn site (Cambridgeshire and Suffolk) grow large quantities of outdoor lettuce between March and October (∼65% of total UK production (Norman 2025)), when *N. ribisnigri* is feeding and reproducing on its secondary host plants (including lettuce). It is possible that the observed differences in (*F_st_*) levels in this area are influenced by a combination of the availability of outdoor lettuce and the limited dispersal rate of *N. ribisnigri*, causing a shift in the local population due to high selection pressures. However, this does not fully explain the differences observed, as outdoor lettuce is cultivated in the wider area of the East. Other cultivation practices, such as the application of insecticides (Bass et al. 2014, Arias et al. 2019), could also be accounting for the observed differences in *N. ribisnigri* at Broom’s Barn. It has been demonstrated that insecticides can significantly impact aphid genetics, driving the evolution of resistance and altering genetic diversity, including changing the genetic structure and composition of aphid populations (Thomas et al. 2016, Hawkins et al. 2019).

We have shown that western *N. ribisnigri* populations can migrate to the East, though this pattern is not reversed, possibly because of the dominance of the summer host, grown commercially in the East, and potentially the influence of the prevailing southwesterly winds, which have been shown to closely match aphid migration (Hu et al. 2016). It was noted that both Writtle and Wye (both situated in the East of England) had several individuals from the West populations, whereas Rothamsted and Kirton (also situated in the East) had very few (Fig. 4). The low number of West individuals in both Rothamsted and Kirton could be an artifact of the low sample number or the lack of suitable primary host plants, creating an ecological barrier (Vialatte et al. 2005). Many different barriers exist that can result in population isolation, limit genetic exchange, and cause bottlenecks. A study on Russian wheat aphid (*Diuraphis noxia*) found that clear geographical isolation existed between populations in the North and South of China, with gene flow interrupted by desert regions (Zhang et al. 2012). For *N. ribisnigri*, it is possible that the limited genetic exchange between the East and the West is a result of ecological processes, rather than physical barriers. An ecological differentiation between the use of cultivated and wild host plants could account for the divide between *N. ribnisnigri* populations in the East and West of England. Since much of the secondary host plant, lettuce, is cultivated in the eastern side of the United Kingdom, this is likely one of the components driving a shift in *N. ribisnigri* population genetics. Additionally, the low numbers caught throughout the United Kingdom, combined with a high inbreeding coefficient, suggest they either exist in lower numbers in the wild or disperse (migrate) at a lower rate compared to more polyphagous aphids. Similar results have been found for other aphids such as the pea aphid (*Acyrthosiphum pisum*) and English-grain aphid (*S. avenae*), which are both highly polyphagous species (Via and Hawthorne 2002, Vialatte et al. 2005). For example, use of microsatellite markers showed that with populations of English-grain aphids collected from both cultivated and uncultivated host plants within the same area, limited genetic exchange occurred between populations (Vialatte et al. 2005). A more recent study highlights that the host plants, along with insecticides, shape the evolution of genetic and clonal diversity in *M. persicae* (Roy et al. 2022). These findings show that 3 out of 4 genetic clusters have a strong association with host plants, which supports the findings in the present paper.

*N. ribisnigri*, with its comparatively small population size, is likely to have unique ecological specialisms because of its low dispersal rate and host plant availability and is therefore more likely to have its own niche and diet breadth compared to other host-alternating cosmopolitan species such as *Myzus persicae* and *Sitobion avenae* (Blackman 1974, Blackman and Eastop 2000, Dixon and Kindlmann 2020). Additionally, it has been shown that *N. ribisnigri* also produces less winged aphids (alates), compared to other aphid species, and is likely an additional factor in the low catch rates in the 12.2 m RIS suction traps (Diaz and Fereres 2005). The known host range of *N. ribisnigri* covers species of *Ribes* during the winter and members of the Asteraceae (which include lettuce) in the spring and summer. Although there are numerous species from this family in the United Kingdom, *N. ribisnigri* cannot feed and reproduce on all of them (Hough 2013). *Nasonovia ribisnigri* preferentially feed on the young and developing leaves of their host plants and typically do not feed on outer older leaves. This feeding behavior restricts overall aphid capacity per host plant but exploits a host plant niche in which other aphids typically do not exist (Dixon and Kindlmann 1990). It is still unknown why *N. ribisnigri* migrates to the summer host in the spring alongside other host-alternating aphids (both pest and non-pest) yet colonizes outdoor lettuce in late summer. In addition, the unpredictable nature in which *N. ribisnigri* can become a serious pest on outdoor lettuce 1 yr and remain absent the following year further emphasizes the importance of understanding their cryptic autecology.

In conclusion, the use of microsatellite markers has enabled the identification of the previously unknown population structure of a pest of outdoor lettuce in England. Due to the low representative sample size for some years and locations, it has likely resulted in large within-group variation. The analysis has revealed 2 distinct clusters (East and West), with the potential of York being a third grouping, likely to be influenced by the availability of cultivated outdoor lettuce as a summer host. Time series analysis showed little genetic difference and therefore no evidence for long-term demographic change. This study shows that *N. ribisnigri* populations are predominantly holocyclic, even in the South of England, and that the species overwinters in the egg stage. Despite the subsequent breakdown of host plant resistance in cultivated lettuce (*Nr-*gene) in 2009 (in the United Kingdom) and strong selection pressures due to the application of insecticides, a demographic change was not observed. This indicates that these selection pressures and environmental changes have little effect on the genetic diversity of *N. ribisnigri*. However, a demographic change was observed at one site in the East of England, which could partly be explained by the high level of production of lettuce and associated practices in that area. In addition, there is evidence for a deficiency of heterozygotes, which could be explained by inbreeding. This supports the hypothesis that the population size of *N. ribisnigri* in England is small, as suggested by the low numbers of individuals captured in the suction traps (RIS 2022). These results have implications for future management strategies for this species, although further studies are required to fully understand its population dynamics, such as understanding the cryptic autecology and potential use of intermediate non-crop summer hosts.

## Supplementary Material

ieaf082_Supplementary_Data

## Data Availability

The data set used in these analyses will be published with a DOI on the Rothamsted Research Repository (https://repository.rothamsted.ac.uk/) under a Creative Commons Attribution 4.0 License. For the purposes of open access, the authors have applied a Creative Commons Attribution (CC-BY) license to any author-accepted manuscript version arising.
