# Supplementary material for: Population genetics show that aphids (Hemiptera: Aphididae) are limited by summer host-plant distribution at the regional scale
Source: J Insect Sci. 2025 Oct 6;25(5):ieaf082. doi: 10.1093/jisesa/ieaf082 (PMC12499771; doi:10.1093/jisesa/ieaf082)
Supplement: ieaf082_Supplementary_Data [file ieaf082_supplementary_data.zip › Supplementary Material 31JUL25.docx]

**Tables**

**Table S1.** Msatcommander search parameters for microsatellite discovery and the number of microsatellites discovered and passing initial Primer3 parameters.

| **Motif** | **Minimum no. of repeats** | **Microsatellites discovered** |
| --- | --- | --- |
| Di-nucleotide | 8 | 25 |
| Tri-nucleotide | 5 | 36 |
| Tetra-nucleotide | 4 | 5 |
| Penta-nucleotide | 4 | 1 |
| Hexa-nucleotide | 4 | 1 |
| Total | - | 68 |

**Table S2.** *Nasonovia ribisnigri* cultures on *Lactuca sativa* that were used for microsatellite validation.

| ***N*. *ribisnigri* culture** | **Host plant** | **Culture origin** |
| --- | --- | --- |
| Nr4 | Pinokkio (S) | Chichester, 1999 |
| Nr8 | Pinokkio (S) | York, 1999 |
| Nr29 | Pinokkio (S) | Suffolk, 1999 |
| 4850a | Pinokkio (S) | Lincolnshire, 2003 (**Hough, 2013**) |
| WT Kent | Pinokkio (S) | Kent, 2010 (**Hough, 2013**) |
| Kent CL | Pinokkio (S) | Kent, 2009 (**Hough, 2013**) |
|  |  |  |

| **Locus** | **Motifs repeat** | **Primers (5’ -> 3’)** | **Ta(°C)** | **Size range (bp)** | **N_A_** | ***H_O_*** | ***H_E_*** |
| --- | --- | --- | --- | --- | --- | --- | --- |
| **Nrib7** | (AT)10 | F: CACGACGTTGTAAAACGACTCCGGACATCTTCTTCGCGT  R: TCGACTTGACCCGATTCGC | 50 | 160-175 | 9 | 0.61 | 0.75 |
| **Nrib15** | (AG)15 | F: CACGACGTTGTAAAACGACTCAACAGTCAACATCCCCAGTGT  R: TAGTCCACGCCCAACTGCAT | 50 | 125-140 | 21 | 0.47 | 0.85 |
| **Nrib26** | (AGC)5 | F: CACGACGTTGTAAAACGACTGCCTTTCTCGTACTGTGTATCG  R: ACCCTTGACGAAAAGAAAACACG | 58 | 182-196 | 12 | 0.37 | 0.79 |
| **Nrib30** | (AAT)5 | F: CACGACGTTGTAAAACGACTTTGGATATGAGACGAGCGGC  R: GGCAAGTTTAAGGGCGACGG | 50 | 175-186 | 4 | 0.16 | 0.66 |
| **Nrib31** | (AAT)6 | F: CACGACGTTGTAAAACGACAGCTGTTTTAATGCGTGCGACA  R: GATCCCTATCTCCGTCCCCG | 58 | 192-205 | 6 | 0.30 | 0.57 |
| **Nrib32** | (AAT)5 | F: CACGACGTTGTAAAACGACGGTGGTGGTGAGGAAGAGCA  R: CACGTGCAGGTCGAGTTCAA | 62 | 180-188 | 10 | 0.46 | 0.75 |
| Nrib48 | (AAG)5 | F: CACGACGTTGTAAAACGACAGGTTTTGTGTTGGCAGCGA  R: AGGCAAGGTATATCGGCAGACC | 60 | 144-162 | - | - | - |
| Nrib49 | (AAC)5 | F: CACGACGTTGTAAAACGACCTTTTGCCTTGGCGTCCGAC  R: AATACACCACCGCCACCACC | 62 | 140-156 | - | - | - |
| **Nrib51** | (AAT)6 | F: CACGACGTTGTAAAACGACTGGCGTATGTTTTCGTGGGT  R: CCCACTTGCCCACCTAGCTT | 58 | 120-134 | 9 | 0.41 | 0.59 |
| Nrib53 | (ACG)5 | F: CACGACGTTGTAAAACGACGCGCAGAGATTTTCCCGTCG  R: ACCTGATCATCGGTCACACAAC | 58 | 130-143 | - | - | - |
| Nrib58 | (AAT)6 | F: CACGACGTTGTAAAACGACTAAATGGGCGTCGGGTTGGA  R: GGGTAACTGGCTCGATCGCT | 62 | 104-118 | - | - | - |
| **Nrib59** | (AAT)6 | F: CACGACGTTGTAAAACGACACCACAGTTATCCGAGTCCAGA  R: ATTCAACTCAAACGCGCTCTAGA | 58 | 108-120 | 10 | 0.39 | 0.47 |
| Nrib65 | (ACAT)4 | F: CACGACGTTGTAAAACGACGGCACGTCGACAGAACCCT  R: TCTGCCGGTCGTCCCTTATTT | 60 | 114-130 | - | - | - |
| Nrib66 | (AAAG)7 | F: CACGACGTTGTAAAACGACGCAGGCCCCGTAATTAACAGC  R: CCGTCTCTCCCCTTGCACTC | 62 | 167-182 | - | - | - |
| Nrib67 | (ATATT)4 | F: CACGACGTTGTAAAACGACTGAGTATTTGCCGCCGGTCT  R: CCGCTCGAGTGTGTGTACGA | 62 | 161-177 | - | - | - |

**Table S3**: The 15 identified and developed microsatellites for *Nasonovia ribisnigri* and their F/R primers sets. Highlighted in bold are the eight microsatellites used for this study. Ta(°C): Annealing temperature; N_A_: Number of alleles, *H_O_* –observed heterozygosity, *H_E_* –expected heterozygosity.

**Table S4.** The fluorescent dye parameters used for microsatellite analysis.

| **Trace** | **Expected no. of peaks** | **Repeat units** | **Range Start** |
| --- | --- | --- | --- |
| 6-FAM | 2 | 4 | 160-450 |
| VIC | 2 | 4 | 200-280 |
| NED | 2 | 4 | 200-320 |
| PET | 2 | 4 | 140-480 |

**Wellesbourne**

**Wellesbourne**

**Preston**

**Preston**

**Kirton II**

**Kirton II**

**Broom’s Barn**

**Broom’s Barn**

**Rothamsted**

**Rothamsted**

**Writtle**

**Writtle**

**Hereford**

**Hereford**

**Starcross**

**Starcross**

**Wye**

**Wye**

**York**

Figure 5.1: The Rothamsted Insect Survey (RIS) suction trap sites throughout the UK that were used in the study.**York**

**Figure S1:** The Rothamsted Insect Survey (RIS) 12.2 m suction trap sites throughout the UK.

**Figure 2**


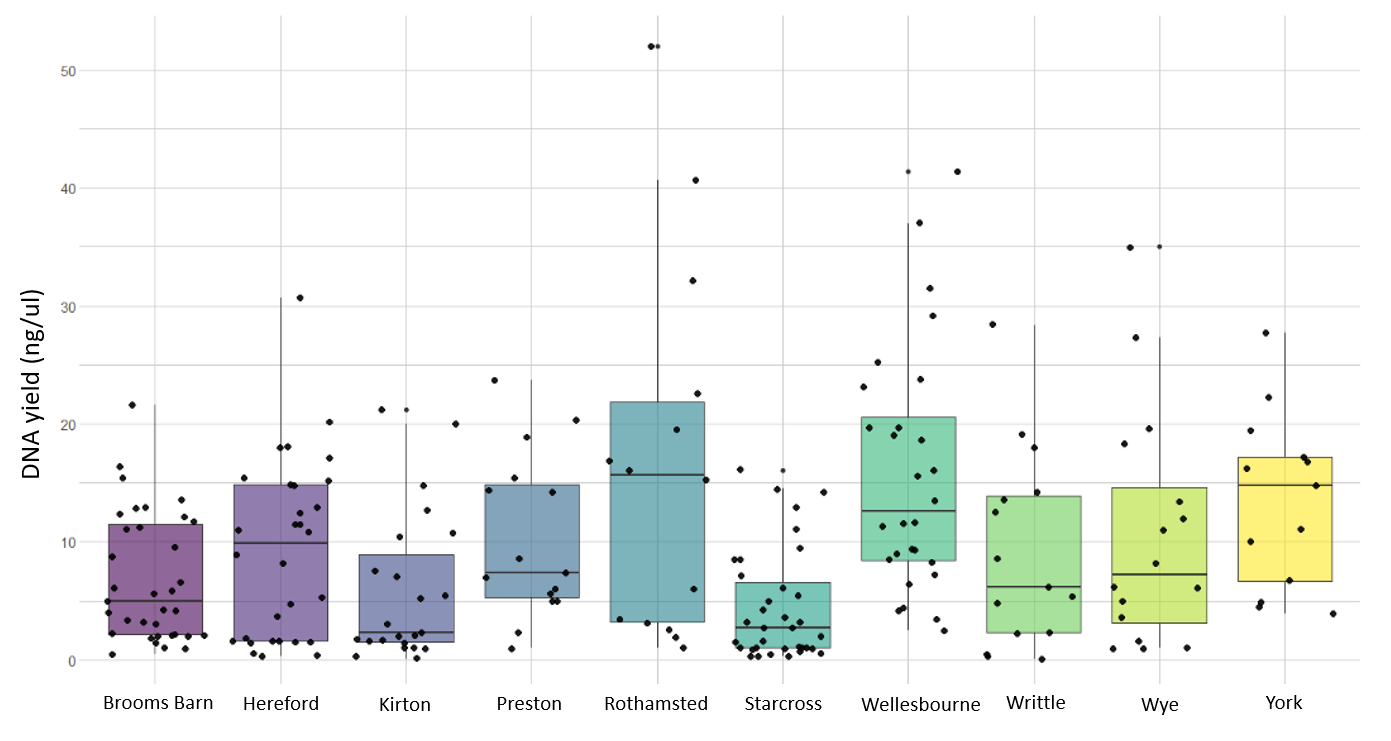


**Figure S2:** The Rothamsted Insect Survey (RIS) 12.2 m suction trap sites throughout the UK. The red line indicates the cut-off of 4 µl/ng in which samples were too low to proceed with analysis.


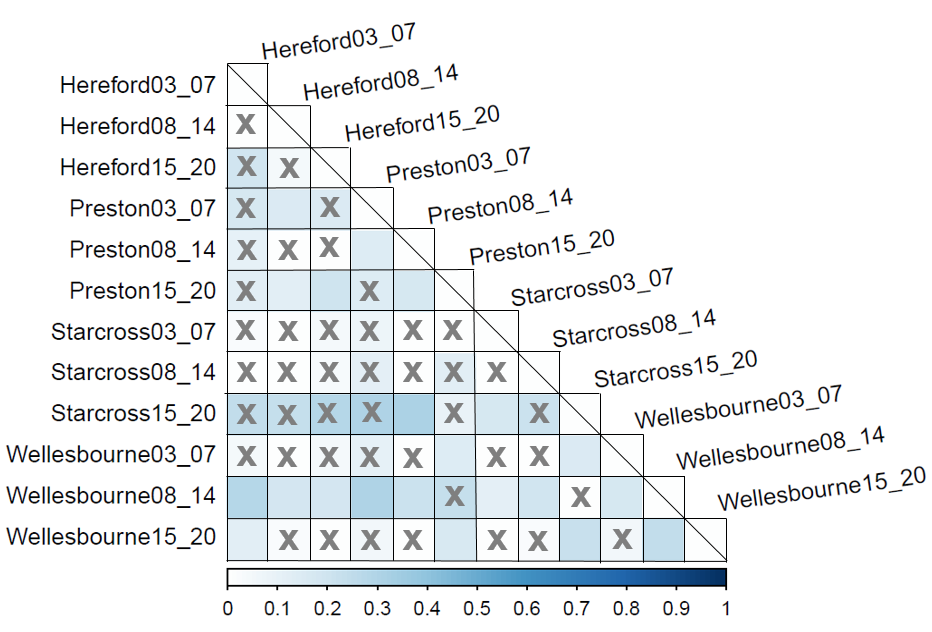

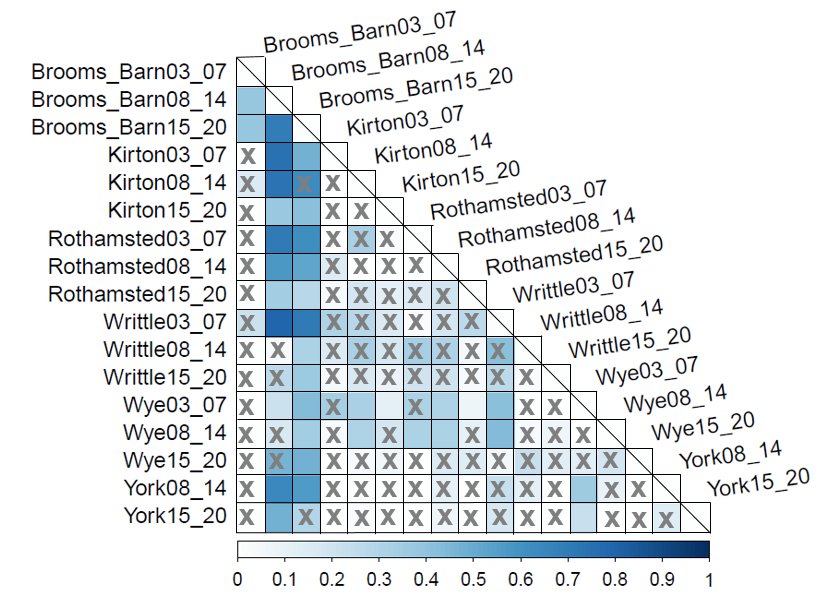


**B)**

**A)**

**Figure S3:** Population pairwise *F_ST_* of *Nasonovia ribisnigri* between population sites between 2003-2020 showing the level of genetic differentiation between the East (A) and the West (B). 03_07 = 2003-2007; 08_14 = 2008-2014; 15_20 = 2015-2020. The scale bar on the x axis highlights the pairwise *F_ST_* value, the darker the square, the higher the *F_ST_* value. X indicates no significant *F_ST_* value between two populations (significance set to *F_ST_* >0.20, *P* = < 0.05).
